# Hospital length of stay and 30-day readmissions in older people: their association in a 20-year cohort study in Italy

**DOI:** 10.1186/s12877-023-03884-4

**Published:** 2023-03-21

**Authors:** Caterina Trevisan, Marianna Noale, Giancarlo Zatti, Davide Liborio Vetrano, Stefania Maggi, Giuseppe Sergi

**Affiliations:** 1 Geriatric Unit, Department of Medicine, University of Padova, Padua, Italy; 2 Department of Medical Sciences, University of Ferrara, Via Aldo Moro, 8, Ferrara, 44124 Italy; 3 Aging Research Center, Department of Neurobiology, Care Sciences and Society, Karolinska Institutet and Stockholm University, Stockholm, Sweden; 4 Neuroscience Institute, National Research Council, Padua, Italy; 5 Stockholm Gerontology Research Center, Stockholm, Sweden

**Keywords:** Hospitalization, Hospital readmission, Length of stay, 30-day readmission

## Abstract

**Background:**

There are conflicting data on whether hospital length of stay (LOS) reduction affects readmission rates in older adults. We explored 20-year trends of hospital LOS and 30-day rehospitalizations in a cohort of Italian older people, and investigated their association.

**Methods:**

Participants in the Pro.V.A. project (*n* = 3099) were followed-up from 1996 to 2018. LOS and 30-day rehospitalizations, i.e. new hospitalizations within 30 days from a previous discharge, were obtained from personal interviews and regional registers. Rehospitalizations in the 6 months before death were also assessed. Linear regressions evaluated the associations between LOS and the frequency of 30-day rehospitalizations, adjusting for the mean age of the cohort within each year.

**Results:**

Over 20 years, 2320 (74.9%) participants were hospitalized. Mean LOS gradually decreased from 17.3 days in 1996 to 11.3 days in 2018, while 30-day rehospitalization rates increased from 6.6% in 1996 to 13.6% in 2018. LOS was inversely associated with 30-day rehospitalizations frequency over time (β = -2.33, *p* = 0.01), similarly in men and women. A total of 1506 individuals was hospitalized within 6 months before death. The frequency of 30-day readmissions at the end of life increased from 1.4% in 1997 to 8.3% in 2017 and was associated with mean LOS (β = -1.17, *p* = 0.03).

**Conclusions:**

The gradual LOS reduction observed in the latter decades is associated with higher 30-day readmission rates in older patients in Italy. This suggests that a careful pre-discharge assessment is warranted in older people, and that community healthcare services should be improved to reduce the risk of readmission.

**Supplementary Information:**

The online version contains supplementary material available at 10.1186/s12877-023-03884-4.

## Background

Older people represent a large share of in-hospital patients, which is expected to grow even more in consideration of the steep aging of our societies. The Italian Ministry of Health reported in 2019 that older individuals presented with the highest hospitalization rates in the country, ranging from 156.6 to 255.48 per 1000 inhabitants for the categories of 65–74 and ≥ 75 years, respectively [1]. The burden of these events on the healthcare systems is even heavier considering that older age is associated with longer hospital stays [2] and more frequent 30-day readmissions [3, 4]. Recent reports show that 30-day rehospitalization rates in the general population range between 10 and 20% [3–7], with nearly half of the readmissions deemed as potentially preventable [8, 9].

In this context, specific thresholds for the length of stay (LOS) and rate of readmissions [10, 11] have been proposed as quality indicators of care to enhance the efficacy and sustainability of acute care services. However, these goals appear harder to be reached when dealing with geriatric patients due to the frequent coexistence of acute and chronic diseases, functional impairments, and social disadvantages that generate complex clinical pictures and challenge their early discharge and permanence in the community. In most vulnerable patients, any LOS reduction not due to limiting discharge delays for non-medical reasons may lead to premature discharge with unsolved clinical or social issues, determining a higher risk of readmissions [12].

The relationship between LOS and readmission rate has been evaluated in previous observational studies drawing inconsistent conclusions [4, 5, 13–17]. On the other hand, scarce evidence has emerged on the possible association between changes in these phenomena over time, in particular in older people.

This study aimed to explore the trends and relationship between LOS and 30-day rehospitalizations (all, and in the 6 months prior to death) in older people, from 1996 to 2018. The hypothesis is that LOS reduction in the older population may be associated with a higher rehospitalization rate.

## Methods

### Study population

The Progetto Veneto Anziani (Pro.V.A.) consists of an observational cohort study conducted in two cities of the Veneto Region (northeastern Italy), with a baseline assessment between 1995 and 1997 and two active follow-up assessments, after a mean of 4.4 and 7 years from the baseline, respectively [18]. A passive follow-up based on administrative data from the regional electronic health records allowed to obtain information on participants’ vital status and hospitalizations until December 31^st^, 2018 [19, 20].

### Data collection

The Pro.V.A. study population included a random sample of 3,099 individuals aged ≥ 65 years, both community-dwelling and institutionalized subjects, identified considering a multistage stratified sampling method. At baseline and the 4.4- and 7-year follow-ups, participants were evaluated through face-to-face interviews, medical records evaluation, standardized questionnaires, and physical examinations by trained physicians and nurses. From these assessments, we collected sociodemographic information (age, sex, years of schooling, living arrangements categorized as living with somebody, living alone, and living in a nursing home) and data on lifestyle (smoking and drinking habits, and Body Mass Index [BMI] from the measurement of body weight and height) and functional status (self-sufficiency in eating, bathing, toileting, dressing, moving, and urinary/fecal continence, through the Katz index [21]). As concerns chronic diseases, for this study, we considered the following conditions: cardiovascular diseases (CVD, including congestive heart failure, coronary artery diseases, or peripheral artery disease), osteoarticular diseases (including osteoporosis, history of hip fracture, osteoarthritis, or rheumatoid arthritis), neurological diseases (including stroke, Parkinson’s disease, cognitive impairment), diabetes, hypertension, dyslipidemia, chronic obstructive pulmonary disease (COPD), chronic kidney disease, vision deficits, hearing deficits. Moreover, the presence of depressive symptoms was defined as a score > 10 at the Geriatric Depression Scale [22]. Fall history was evaluated by asking the participants whether they had fallen in the past year. Finally, frailty, defined according to the criteria of Fried and colleagues, was assessed through physical tests and validated scales. In particular, we considered as frail the individuals who presented three or more of the following items: low gait speed, low physical activity, weakness, weight loss, and exhaustion [23]. Details on frailty assessment in the Pro.V.A. study can be found in previous publications [24]. Briefly, slowness was evaluated based on the 4-m walking test at the usual pace, and the lowest sex- and height-specific quintile was taken as a cutoff. To assess low physical activity, we estimated daily energy expenditure from a structured questionnaire on the frequency of various activities and considered the lowest sex-specific quintile of that value as a cutoff. As concerns weakness, we measured participants’ handgrip with a dynamometer, twice for each hand, and used the lowest sex- and BMI-specific quintile of handgrip as a threshold. Weight loss was investigated by asking to the participants if they had experienced a reduction in body weight ≥ 5 kg in the last year. Finally, exhaustion was defined based on the self-reported feeling of lack of energy and a GDS score ≥ 11 points.

### Outcomes

Data on hospitalizations were collected through structured interviews performed by trained medical personnel for the period from baseline to 1997 (due to unavailability of registers data in that timeframe), thereafter from administrative electronic health records. Based on such information, we computed the LOS of all hospitalization episodes (including also possible rehospitalizations) as the time window, in days, from admission to discharge. Rehospitalizations were defined as new hospitalizations within 30 days from a previous discharge [6, 25]; planned hospitalizations, hospital stays with a duration < 2 days, and transfers from one hospital ward to another on the same day of the discharge were not considered as rehospitalizations. Rehospitalizations in the 6 months preceding death were also assessed. Cause of hospitalizations were obtained through linkage from regional registers considering the ICD-9 codes reported at discharge as primary diagnosis.

### Statistical analysis

The characteristics of the study participants were summarized as means ± standard deviation (SD) or median (25th-75th percentile) for quantitative measures and as counts and percentages for categorical variables. The comparison of these characteristics by sex was performed through chi-square or Fisher exact test for categorical variables and generalized linear models or Wilcoxon sum rank test for quantitative variables.

Moving averages, a method for smoothing time series by averaging a fixed number of consecutive terms, were also calculated for the variables related to mean LOS, the percentage of 30-day rehospitalizations and 30-day rehospitalizations in the 6 months before death. A window size of 3 for the variable “year of hospitalization” was considered as number of consecutive terms to calculate moving averages.

Linear regression models, with data evaluated by year of hospitalization, were defined to examine the associations of the mean duration of hospitalizations (independent variable) with (a) the percentage of 30-day rehospitalizations, and (b) the percentage of 30-day rehospitalizations in the 6 months before death (dependent variables), adjusting for the mean age of the cohort in the considered year. The regression models considered data evaluated by year of hospitalization (statistical unit), so both the dependent variables and the independent variables had 20 values; age (mean age of the cohort) and LOS (mean LOS for the cohort) were assumed to be constant within each year considered as statistical unit in the models. Linear regression assumptions (linearity of the relationships between predictors and outcome; normality of the errors; homogeneity of variances of the error; independence of the errors; influence and collinearity of predictors) were preliminarily evaluated.

Analyses were repeated considering men and women separately, and moving averages. In light of the differences in the assessment of the hospitalizations in the first years of observation, we performed a sensitivity analysis considering only the period from 1997 to 2018. Secondly, in light of the trends of LOS and rehospitalizations over time, we repeated the linear regression analyses after splitting the observation period into two 10-year time frames. A third sensitivity analysis was performed after excluding those living in a nursing home. Finally, to explore the association between LOS and rehospitalizations in light of participants’ changes in sociodemographic and functional status, whose data were available at baseline and after 4 and 7.7 years, we ran models adjusted for age, living arrangements, and ADL scores (as time-varying variables), from 1996 to 2006.

All statistical tests were two-tailed and statistical significance was assumed for a *p*-value < 0.05. The analyses were performed using SAS, V.9.4 (SAS Institute, Cary, NC).

## Results

For the present analysis, we considered an analytical sample of 3,099 subjects. At baseline (Table 1), participants’ age was 76.3 ± 7.8 years and 1244 (41%) were men; almost half of the sample had completed at least five years of education (51.2%), and 521 (17.2%) lived alone. The most frequent chronic conditions were hypertension (75%), osteoarticular diseases (66%), chronic kidney diseases (31%), CVD (29%), and neurological diseases (16%). Almost 8% of the sample was frail, with no differences by sex, and one out of three individuals had experienced at least one fall in the past year, with a higher prevalence reported by women compared to men.

**Table 1:** Characteristics of the study population at the baseline

	**Overall** **(*n* = 3099)**	**Men** **(*n* = 1244)**	**Women** **(*n* = 1855)**	***P*-value**
Age, years, mean ± SD	76.3 ± 7.8	76.4 ± 7.9	76.3 ± 7.8	0.263
Education ≥ 5 years, n (%)	1586 (51.2)	821 (66.0)	765 (41.3)	< 0.0001
Monthly income > 500€, n (%)	1175 (39.0)	629 (51.1)	546 (30.6)	< 0.0001
Living alone, n (%)	521 (17.2)	107 (8.7)	414 (23.0)	< 0.0001
Living in nursing home, n (%)	118 (3.8)	25 (2.0)	93 (5.1)	< 0.0001
No current smoking, n (%)	2814 (91.2)	1045 (84.2)	1769 (96.0)	< 0.0001
No current drinking, n (%)	2735 (88.3)	912 (73.3)	1823 (98.3)	< 0.0001
BMI, kg/m^2^, mean ± SD	27.8 ± 4.6	26.8 ± 3.8	28.1 ± 5.0	< 0.0001
ADL score, mean ± SD	4.8 ± 1.6	4.8 ± 1.6	4.8 ± 1.5	0.478
Vision impairment, n (%)	790 (25.0)	317 (25.5)	473 (25.5)	0.992
Hearing impairment, n (%)	1748 (56.4)	803 (64.6)	945 (50.9)	< 0.0001
CVD, n (%)	882 (28.5)	415 (33.5)	467 (25.2)	< 0.0001
Osteoarticular diseases, n (%)	2025 (65.5)	601 (48.5)	1424 (76.9)	< 0.0001
Neurological diseases, n (%)	497 (16.1)	190 (15.3)	307 (16.6)	0.349
Diabetes, n (%)	309 (10.0)	100 (8.0)	209 (11.3)	0.003
Cancer, n (%)	245 (7.9)	111 (8.9)	134 (7.2)	0.086
COPD, n (%)	300 (9.7)	205 (16.5)	95 (5.1)	< 0.0001
Hypertension	2304 (74.5)	912 (73.4)	1392 (75.2)	0.252
Dyslipidemia, n (%)	181 (5.8)	54 (4.3)	127 (6.9)	0.004
Chronic kidney disease, n (%)	957 (31.2)	261 (21.2)	696 (38.0)	< 0.0001
Depressive symptom, n (%)	1101 (35.5)	334 (26.9)	767 (41.4)	< 0.0001
Fall in the previous year, n (%)	951 (30.7)	308 (24.8)	643 (34.7)	< 0.0001
Frailty, n (%)	243 (7.8)	88 (7.1)	155 (8.4)	0.166

*n* = 54 and *n* = 10 individuals had missing information on frailty and falls, respectively

*Abbreviations: ADL* Activities of daily living, *BMI* Body mass index, *COPD* Chronic obstructive pulmonary disease, *CVD* Cardiovascular diseases

During the 20-year follow-up, 2320 (74.9%) participants experienced at least one hospitalization; the mean number of hospitalizations was 4.3 ± 4.7, and the mean LOS for each hospitalization (also considering possible rehospitalizations) was 14.0 ± 17.0 days. Women were more likely to be hospitalized at least once (76.1% vs 73.0%, *p* = 0.049) and experienced longer LOS (median 11 vs 10 days, *p* = 0.029). The most frequent hospitalization’s underlying causes, according to the ICD-9 codes of the primary diagnosis at discharge, were CVD, followed by respiratory, gastrointestinal, nervous diseases and injuries/poisonings. Over time, we observed a gradual increase in hospitalizations due to respiratory diseases or injuries/poisoning, while those for neurological causes decreased (Supplementary Table 1).

**Fig. 1:**
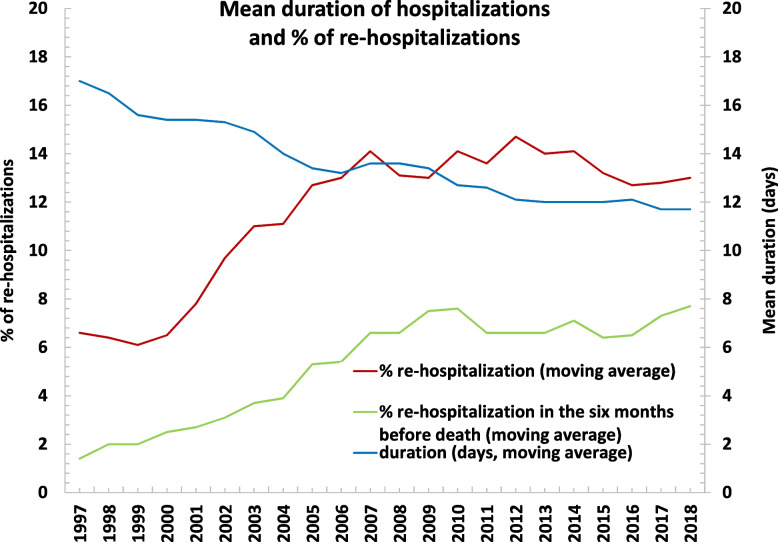
Mean duration of hospitalization (days) and percentages of rehospitalization (overall and in the six months before death), by year of hospitalization

As reported in Table 2, over time, we found a significant decreasing trend in mean LOS, ranging from 17.3 days in 1996 to 11.3 days in 2018, and median LOS, from 10.3 days in 1996 to 9.5 days in 2018 (*p* < 0.001 according to Jonckheere-Terpstra test). The reduction of LOS was more marked when considering the mean than the median values, and a parallel decrease in the LOS standard deviations was noted over time. Conversely, the frequency of 30-day unplanned rehospitalizations during the same period increased from 6.6% in 1996 to 13.6% in 2018 (Fig. 1). In a linear regression model adjusted for the mean age of the cohort, considering year as a statistical unit, the mean LOS was inversely associated with the frequency of 30-day rehospitalizations (β = -2.33, SE 0.66, *p* = 0.01; R^2^adj = 0.62). This relationship was stronger in the first ten years of observations (Supplementary Table 2). Results were substantially confirmed considering moving averages (β = -2.68, SE 0.50, *p* = 0.001; R^2^adj = 0.82). When stratifying the analyses by sex, results seemed to be more marked for men (β = -1.39, SE 0.82, *p* = 0.11) than women (β = -0.78, SE 0.86, *p* = 0.376). Results did not change after excluding institutionalized individuals. Moreover, a similar but non-significant trend (likely due to the few number of observation) was found after adjusting also for living arrangements and functional status considering only the first 10 years of observation (from 1996 to 2006; β = -0.30, SE = 1.24, *p* = 0.83).

**Table 2:** Average length of stay per admission (considering only participants with at least one hospitalization during follow-up) and 30-day rehospitalization, from 1996 to 2018

**Year**	**Cohort size** **(n)**	**Participants** **hospitalized** **(n)**	**Length of stay**		**30-day rehospitalization**^a^ **n (%)**	**30-day** **rehospitalization**^a^ **in the 6 months** **before death n (%)**
			**Mean ± SD**	**Median** **(Q1, Q3)**		
1996	3045	145	17.3 ± 32.7	10.3 (6.6, 15.8)	-	-
1997	2923	561	16.5 ± 21.7	11 (7.1, 18)	37 (6.6)	8 (1.4)
1998	2774	494	15.3 ± 16.8	11 (7.2, 16.6)	30 (6.1)	13 (2.6)
1999	2611	560	14.9 ± 14.5	11 (7.3, 16.2)	32 (5.7)	12 (2.1)
2000	2468	590	15.1 ± 14.5	11 (7.6, 16.7)	46 (7.8)	16 (2.7)
2001	2291	561	15.4 ± 15.6	11.3 (7.8, 17)	55 (9.8)	18 (3.2)
2002	2146	532	14.4 ± 13.3	11.2 (7.3, 15.9)	61 (11.5)	18 (3.4)
2003	1998	510	14.0 ± 13.6	11 (7.6, 15.6)	60 (11.8)	23 (4.5)
2004	1873	437	13.1 ± 12.3	10.8 (7.5, 15.6)	44 (10.1)	17 (3.9)
2005	1741	428	12.9 ± 11.8	10.2 (7.3, 15.1)	69 (16.1)	32 (7.5)
2006	1617	392	13.6 ± 12.6	11 (7.5, 15.6)	50 (12.8)	19 (4.9)
2007	1495	375	14.0 ± 13.9	11 (7.8, 15.8)	50 (13.3)	28 (7.5)
2008	1356	358	12.9 ± 12.1	10.5 (7.3, 15.4)	47 (13.1)	27 (7.5)
2009	1238	309	13.1 ± 12.4	10.7 (7.4, 15)	39 (12.6)	23 (7.4)
2010	1131	264	11.8 ± 8.2	10 (7, 14.7)	44 (16.7)	21 (8.0)
2011	1020	258	12.8 ± 9.9	10.5 (6.8, 14.8)	30 (11.6)	11 (4.3)
2012	931	216	11.6 ± 9.1	9.6 (6.8 ± 13.4)	34 (15.7)	16 (7.4)
2013	839	211	11.7 ± 9.2	10.3 (6.9, 13.5)	31 (14.7)	17 (8.1)
2014	751	191	12.6 ± 10.0	10.3 (7, 14.3)	23 (12.0)	11 (5.8)
2015	665	184	11.3 ± 8.9	10.2 (6.9, 13.5)	24 (13.0)	10 (5.4)
2016	591	144	12.0 ± 8.7	10.1 (6.4, 14.4)	19 (13.2)	12 (8.3)
2017	507	156	11.6 ± 8.4	9.8 (6.6, 14.3)	19 (12.2)	13 (8.3)
2018	440	125	11.3 ± 10.1	9.5 (6.4, 13.5)	17 (13.6)	8 (6.4)

^a^ Not considering rehospitalization after a day-hospital, planned hospitalizations, and transfers from a hospital ward to another on the same day of the discharge

Among the participants who died during the follow-up (2638, 86%), hospitalizations in the 6 months before death occurred for 1506 (57%). Of these, 614 (40.8%) hospitalizations occurred within 30 days after the previous discharge. The frequency of participants who experienced 30-day readmissions in the 6 months before death increased from 1.4% in 1997 to 8.3% in 2017 (Table 2, Fig. 1, Supplementary Fig. 1; data for 1996 and 2018 were excluded due to the small number of events in those years). Considering this outcome, the mean LOS remained significantly associated with the frequency of 30-day rehospitalizations (β = -1.17, SE 0.49, *p*-value = 0.03; R^2^adj = 0.62). Similar results were obtained after stratification of the analyses by sex and considering only data from 1997 to 2018 (data not shown).

## Discussion

This study shows that the length of hospital stay evaluated following the same cohort of older people decreased over the last 20 years. This reduction was accompanied by a higher frequency of 30-day readmissions, and a similar trend was observed when rehospitalizations occurred at the end of life.

The LOS decrease observed in several countries during the last decades is likely the result of both improvements in healthcare services’ efficiency and interventions to reach established goals for reimbursement or budget expenditure [1, 2, 12, 25]. Interestingly, previous works focused on secular trends showed that the reduction in hospital admissions runs parallel with the decrease in rehospitalization and mortality rates within 30 days from hospital discharge, despite the growing complexity of in-hospital patients [25, 26]. However, one of these studies did not consider changes in LOS [26], while, in another work, the subgroup analysis on older people showed substantial differences in LOS and readmission rates over time [25].

Our findings highlight that the relationship between LOS and readmissions might differ in advanced age. Indeed, older individuals are often characterized by clinical, functional, and social complexity that could affect both hospital LOS and readmission rates. In this regard, previous studies showed that many factors associated with a higher risk of rehospitalization, such as functional impairments, chronic diseases, and use of multiple medications [4, 5, 7, 13, 14, 27, 28], are highly prevalent in advanced age. Moreover, irrespective of the precipitating acute illness, hospital stay can affect *per se* older individuals’ cognitive and physical performance, making them more vulnerable to new health events and hospitalizations [29]. Such risk is further exacerbated by the lack of adequate community and long-term care services able to cope with patients’ newly developed needs [30].

Overall, this suggests that managing older patients’ complexity and ensuring their safe discharge requires time and that a personalized approach should be implemented. In our study, the marked reduction of the mean and variability of LOS over time indicates that the decreasing trend concerned mainly the longest hospitalizations. However, the patients more likely to deserve a prolonged hospital stay are generally those with greater clinical vulnerability and/or poorer social assistance [31–33]. In these cases, reducing LOS with no concurrent improvements in the social and health services supporting the management of older adults after discharge could have unexpected and detrimental consequences. In line with this hypothesis, we found that the LOS shortening over the last 20 years was associated with an increased frequency of 30-day readmissions. A similar trend was also noted for the 30-day readmissions six months before death. This finding suggests that reducing LOS could also prevent physicians from detecting the most vulnerable patients or organizing a safe discharge to manage their severe conditions at home.

Of note, the readmission rates seemed to increase substantially during the first ten years of observation and then stabilized for the last years of observation. Although multiple factors might have influenced this figure, it is worth noting that the National Health Programme for the years 2006–2008 introduced some organizational changes to improve the health care delivery in the community by facilitating the activities and continuity of care of general practitioners [34]. Accordingly, a recent systematic review found that interventions to reduce readmission risk should include planning the discharge early at the hospital, providing multidisciplinary and personalized services, and enhancing the continuum between hospital and community care [35]. This is particularly evident for the frailest patients, as suggested by our findings on the 30-day readmissions in the last six months of life. In this regard, nowadays, the World Health Organization and all the major geriatric societies underline the need to provide as many healthcare services and assistance as possible at home for older adults. Home health care can prevent avoidable hospital admissions, reduce the need for institutionalization, support caregivers, and improve patients’ satisfaction [36, 37]. These aspects are even more relevant at the end of life since ensuring adequate home care and assistance may allow limiting inappropriate hospitalizations and addressing patients’ preferences, including to die at home [38].

Seemingly in contrast with our results, previous studies found that patients with extended hospital stays had a higher risk of rehospitalization [4, 5, 13, 14]. However, such data reflect just the tendency of individuals with more complex clinical and social conditions to have longer hospital duration and, alongside, a higher probability of being readmitted to the hospital.

The evaluation of trends of average LOS and 30-day readmissions by following the same cohort over time allowed us to provide additional and novel insights. Indeed, although having observed an aging study population for years could be a study limitation, one should have expected that individuals getting older had increased individuals’ complexity and, therefore, longer hospital stays. Instead, the decreasing trend in LOS (which is still longer than the mean LOS for acute hospitalizations in older people in Italy, ranging from 7.7 days in the 65–74 years category to 9.1 days in the 75 + [1]) indicates that health policies may have had a substantial impact on hospital management of older people. To consider the aging of the studied population, linear regression models were adjusted for the mean cohort’s age at each considered year. However, since the participants underwent an active follow-up only during the first years of observation, we could not also take into account changes in living arrangements or functional status until 2018. Future studies should overcome this limitation by disentangling the effects of health policies from that of individuals aging in the relationship between LOS and readmission risk. Other possible weaknesses of our work include the unavailability of data on hospitalizations outside the region of residence of the study participants and on services received by patients in the post-discharge period. The latter issue may be of high interest to evaluate to which extent social and health care after hospital discharge may modify the link between LOS and readmission risk, given that it is well known the importance of post-discharge interventions [30, 39]. Moreover, we did not consider whether the relationship between LOS and readmissions may differ by individuals’ chronic diseases and specific reasons for hospitalization. As recent findings suggested [40, 41], this issue deserves special attention and will be explored in future studies. Finally, we did not take into account the ward where participants were hospitalized, so we could not compare the readmission rates of different medical or surgery units. On the other hand, the strengths of our work lie in the inclusion of a large cohort representative of older adults, and in the long 20-year follow-up, which allowed us to observe possible changes in LOS and readmission rates over time.

## Conclusions

The gradual reduction in the length of hospital stay observed over the last 20 years has also involved older adults and may be associated with higher 30-day readmission rates. Further investigations should explore these findings and disentangle the factors supporting an inverse relationship between LOS and readmission rates. However, in this regard, we corroborate the need for careful pre-discharge assessment and effective community healthcare services to reduce the risk of readmission in older people.

## Supplementary Information

**Additional file 1: Supplementary Figure 1.** Plot of the percentage of rehospitalizations by mean duration of hospitalization (days), by year. **Supplementary Table 1.** Primary diagnosis of hospitalizations for selected years. **Supplementary Table 2. **Linear regression for the association between the length of hospital stay and readmission (all and in the six months before death) in two time periods.

## Data Availability

The data that support the findings of this study are available upon reasonable request from the corresponding author, and with permission of the PI of the Pro.V.A. study.
