# Supplementary material for: Hospital length of stay and 30-day readmissions in older people: their association in a 20-year cohort study in Italy
Source: BMC Geriatr. 2023 Mar 21;23:154. doi: 10.1186/s12877-023-03884-4 (PMC10029164; doi:10.1186/s12877-023-03884-4)
Supplement: Supplementary file 1 — Additional file 1: Supplementary Figure 1. Plot of the percentage of rehospitalizations by mean duration of hospitalization (days), by year. Supplementary Table 1. Primary diagnosis of hospitalizations for selected years. Supplementary Table 2. Linear regression for the association between the length of hospital stay and readmission (all and in the six months before death) in two time periods. [file 12877_2023_3884_MOESM1_ESM.docx]

**SUPPLEMENTARY MATERIAL**

**Supplementary Figure 1.** Plot of the percentage of rehospitalizations by mean duration of hospitalization (days), by year


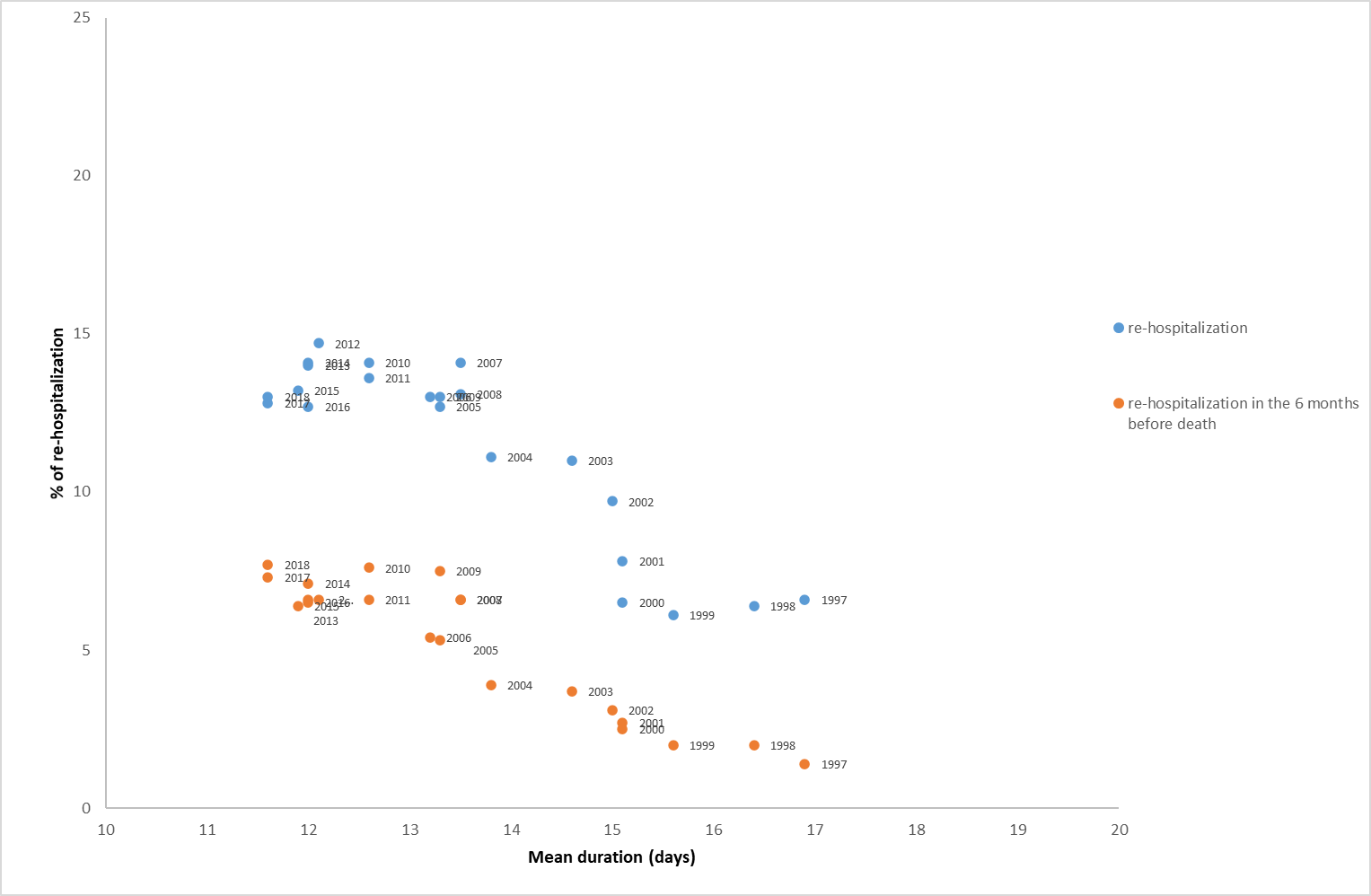


**Supplementary Table 1.** Primary diagnosis of hospitalizations for selected years

| **n hospitalizations (%)** | **1996-2018**  **(n=2320)** | **1996**  **(n=145)** | **1997**  **(n=561)** | **2006**  **(n=392)** | **2007**  **(n=375)** | **2017**  **(n=156)** |
| --- | --- | --- | --- | --- | --- | --- |
| Infectious and parasitic diseases  Neoplasms  Endocrine, nutritional, metabolic diseases  Hematologic diseases  Mental disorders  Neurological diseases  Cardiovascular diseases  Respiratory system diseases  Gastrointestinal diseases  Diseases of the genitourinary system  Dermatological diseases  Diseases musculoskeletal system  Congenital anomalies  Symptoms, signs and ill-defined  Injury and poisoning  Factors influencing health status and contact with health services | 6 (0.3)  694 (29.9)  205 (8.8)  128 (5.5)  132 (5.7)  630 (27.2)  1509 (65.0)  804 (34.7)  618 (26.6)  390 (16.8)  53 (2.3)  362 (15.6)  6 (0.3)  349 (15.0)  630 (27.2)  295 (12.7) | 0 (0.0)  9 (6.2)  3 (2.1)  1 (0.7)  3 (2.1)  14 (9.7)  50 (34.5)  8 (5.5)  6 (4.1)  10 (6.9)  3 (2.1)  13 (9.0)  0 (0.0)  8 (5.5)  11 (7.6)  0 (0.0) | 1 (0.2)  65 (11.6)  28 (5.0)  7 (1.3)  21 (3.7)  90 (16.0)  214 (38.2)  40 (7.1)  54 (9.6)  49 (8.7)  5 (0.9)  38 (6.8)  1 (0.2)  31 (5.5)  37 (6.6)  10 (1.8) | 0 (0.0)  60 (15.3)  9 (2.3)  14 (3.6)  6 (1.5)  18 (4.6)  154 (39.3)  62 (15.8)  52 (13.3)  12 (3.1)  2 (0.5)  28 (7.1)  0 (0.0)  18 (4.6)  38 (9.7)  25 (6.4) | 0 (0.0)  55 (14.7)  12 (3.2)  9 (2.4)  3 (0.8)  17 (4.5)  142 (37.9)  68 (18.1)  48 (12.8)  22 (5.9)  6 (1.6)  22 (5.9)  0 (0.0)  16 (4.3)  44 (11.7)  21 (5.6) | 0 (0.0)  16 (10.3)  2 (1.3)  6 (3.9)  1 (0.6)  3 (1.9)  55 (35.3)  46 (29.5)  16 (10.3)  7 (4.5)  0 (0.0)  4 (2.6)  0 (0.0)  8 (5.1)  24 (15.4)  3 (1.9) |

**Supplementary Table 2. Linear regression for the association between the length of hospital stay and readmission (all and in the six months before death) in two time periods**

|  | **Association of the length of hospital stay with** | |
| --- | --- | --- |
| **Time period** | **30-day rehospitalization** | **30-day rehospitalizations in the 6 months before death** |
| **1996-2006** | β=-2.30, SE=0.65  p=0.008 | β=-1.29, SE=0.29  p=0.002 |
| **2007-2018** | β=-0.66, SE=0.53  p=0.241 | β=-0.10, SE=0.49  p=0.846 |

*Abbreviations*: SE, standard error.
